# Sustainable political commitment is necessary for institutionalizing community participation in health policy-making: Insights from Iran

**DOI:** 10.1186/s12961-024-01111-z

**Published:** 2024-02-13

**Authors:** Maryam Rahbari Bonab, Fatemh Rajabi, Abouali Vedadhir, Reza Majdzadeh

**Affiliations:** 1 Community-Based Participatory Research Center, Tehran University of Medical Sciences, Tehran, Iran; 2 Community-Based Participatory Research Center and University Research and Development Center, Tehran University of Medical Sciences, Tehran, Iran; 3 Department of Anthropology, Faculty of Social Sciences, University of Tehran, Tehran, Iran; 4 Department of Population Health Sciences, Bristol Medical School, University of Bristol, Bristol, UK; 5 School of Health and Social Care, University of Essex, Colchester, UK

**Keywords:** Political commitment, Community participation, Institutionalization, Health policy-making, Iran

## Abstract

**Background:**

Community participation is currently utilized as a national strategy to promote public health and mitigate health inequalities across the world. While community participation is acknowledged as a civic right in the Constitution of Iran and other related upstream documents, the government has typically failed in translating, integrating and implementing community participation in health system policy. The present study was conducted to determine the level of public voice consideration within the health policy in Iran and address fundamental interventions required to promote the public voice in the context of Islamic Republic of Iran (IRI). This study has originality because there is no study that addresses the requirements of institutionalizing community participation especially in low-middle-income countries, so Iran’s experience can be useful for other countries.

**Methods:**

Methodologically, this study utilized a multi-method and multi-strand sequential research design, including qualitative, comparative and documentary studies. In the first phase, the current level of community participation in the health policy cycle of Iran was identified using the International Association for Public Participation (IAP2) spectrum. In the second phase, a comparative study was designed to identify relevant interventions to promote the community participation level in the selected countries under study. In the third phase, a qualitative study was conducted to address the barriers, facilitators and strategies for improving the level of public participation. Accordingly, appropriate interventions and policy options were recommended. Interventions were reviewed in a policy dialogue with policy-makers and community representatives, and their effectiveness, applicability and practical feasibility were evaluated.

**Results:**

Based on the IAP2 spectrum, the level of community participation in the health policy-making process is non-participation, while empowerment is set at the highest level in the upstream documents. Moreover, capacity-building, demand, mobilization of the local population, provision of resources and setting a specific structure were found to be among the key interventions to improve the level of community participation in Iran’s health sector. More importantly, “political will for action” was identified as the driving force for implementing the necessary health interventions.

**Conclusions:**

To sum up, a paradigm shift in the governing social, economic and political philosophy; establishing a real-world and moral dialogue and communication between the government and the society; identifying and managing the conflicts of interest in the leading stockholders of the healthcare system; and, more importantly, maintaining a stable political will for action are integral to promote and institutionalize participatory governance in the health sector of Iran. All of the above will lead us to scheme, implement and institutionalize suitable interventions for participatory governance in health and medicine.

**Supplementary Information:**

The online version contains supplementary material available at 10.1186/s12961-024-01111-z.

## Backgrounds

The significance of mobilizing and maintaining community participation in the health policy cycle was introduced as a key factor in promoting public and community health in the first International Conference on Health Promotion, Ottawa (1986). In this conference, empowerment was recognized as the main factor of success in the attempts of communities to promote health via enabling people to increase control over their health and improve it [1]. Similarly, promoting and improving public participation in the policymaking process and action in social health fields was one of the assertions of the World Conference on Social Determinants of Health (SDH) in 2011. This conference entailed governments to interact with all stakeholders, including the civil society, and mobilize and establish community participation for governing SDH effectively as a necessity [2].

Several terms are interchangeably used with “participation”, such as public, community, and social participation. We use these terms interchangeably for this paper if applicable and define them as follows: direct involvement of citizens or citizen action groups potentially affected by or interested in a decision or action.

Inequality in health is increasing across the world. Meanwhile, strategies such as empowerment, community participation and other bottom-up decision-making approaches are proposed and applied to improve public health and eliminate injustice [3]. Evidence suggests that community participation plays a significant role in promoting health, particularly among the vulnerable and marginalized groups of the society [4]. Participation is also the cornerstone of the human rights framework. The United Nations (UN) Committee on Economic, Social and Cultural Rights considers partnership to be one of the key factors of the right to health [5]. Hence, it is currently applied as part of the national strategy to promote public health [6]. It is also recognized as an important component in the local healthcare service policies and missions [7]. For instance, public participation in the health system of Chile is supported and realized through norms, instructions, operational targets, incentives and financial inputs provided by the Ministry of Health [8]. Likewise, the Thai National Health Assembly (NHA) provides the opportunity for various stakeholder groups to better understand their perspectives through dialogue [9], which plays a key role in utilizing evidence in the policy dialogues [10]. In Brazil, citizen involvement helped to promote a more equitable distribution of public health service [11]. Public health participation is realized through public health conferences held at various levels: local, provincial and national [12], and health councils are permanent and deliberative bodies that formulate, deliberate and control the execution of health policy, including economic and financial aspects[13]. The National Health Conference in France is a consultative institution including 97 independent members and represents a wide spectrum of actors in the health system. Public parties and vulnerable groups, representatives of the patient associations and labour unions are among the independent members of the conference [14].

Iran has gained valuable experience in promoting and sustaining community participation in the health sector over the past half-century, including successful instances of community participation in primary healthcare (PHC). The initial indications of the establishment of the PHC system emerged in 1979, with the full establishment of the primary healthcare network occurring in 1985 [15]. Under the PHC system structure in Iran, every village (sometimes a collective of multiple villages) is equipped with a health house. In larger village settings, including the health house, there is the rural health centre. These health houses serve as the preliminary contact point of the households with the health system. In urban areas, the rural health medical centres provide similar services. This network is managed by the district-level health medical centres, overseen by the country’s medical universities [15, 16].

To promote community participation in Iran, some measures have been taken, including the establishment of local health houses, health workers, house of public participation for health, and provincial and national health assemblies [17]. To address and manage the health issues of urban marginalized groups, the Ministry of Health and Medical Education (MoHME) initially designed and implemented the Health Workers program in the late 1960s. Then, the “Urban Volunteers” program was developed with the aim of promoting public health within the framework of the Health Workers program. Later, it transformed into a national program in 1992 considering its crucial achievements [18]. Similarly, in 2015, the Social Department was incorporated into the organizational chart of the MoHME to promote public health awareness and to coordinate with active non-governmental organizations (NGOs) and charitable organizations within the health sector [19]. One of the key initiatives undertaken by the department was the establishment of provincial and national health assemblies, following the model used in Thailand. In addition, public participation halls were set up in 31 provinces and 296 counties, consisting of 21 representatives from various community groups, tasked with organizing and mobilizing regional populations. However, following the appointment of a new Minister of Health in 2018, the Social Department was removed from the Ministry’s structure. Subsequently, all of the department’s duties and responsibilities were delegated to another subsidiary office within the MoHME. According to experts and key informants, there is a consensus that the level of community participation in Iran’s health system should be enhanced, surpassing the mere provision of services, and it should become an integral part of the policy-making system [20].

The present study was conducted to assess the extent to which the public voice is heard within the health policy-making system, provide a comprehensive view of the state of community participation and address the essential interventions needed to promote the public voice within the pseudo-democratic context of Iran.

This study has originality because, despite the evidence that community participation has an important impact on the sustainability and success of health programs and policies, there are no studies that addresses the requirements of institutionalizing community participation in health systems. Especially in low-middle income countries, so Iran’s experience can be useful for other countries.

## Methods

**Fig. 1:**
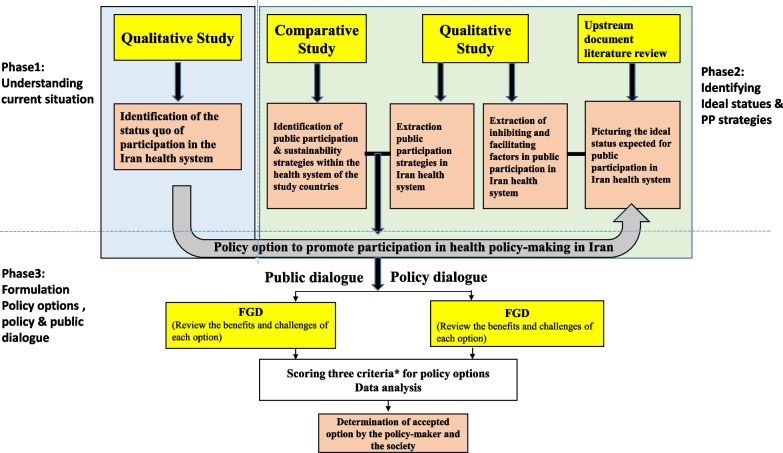
The different phases of this sequential multi-strand study. The yellow box indicates the methodology used, and the orange box the output of each phase. The asterisk (*) indicates the impact on mobilizing public participation in health policy-making”, “operational feasibility” and “acceptance from the perspective of policy-makers and society”

**Fig. 2:**
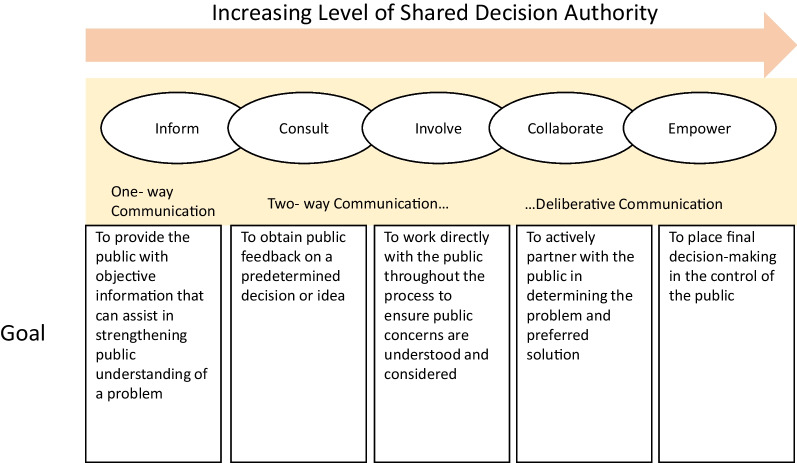
IAP2 Framework: Spectrum of Public Participation

**Fig. 3:**
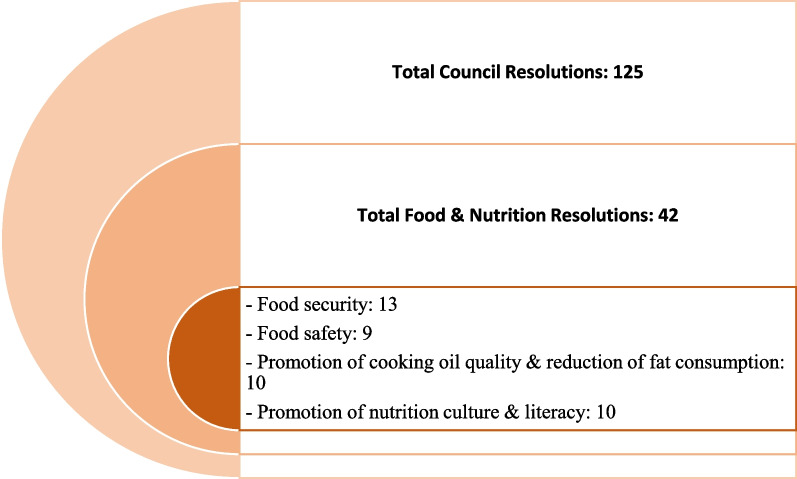
Health and Food Security Supreme Council resolutions of IRI on food and nutrition in 2003–2017

Methodologically, this study utilized a multi-method and multi-strand sequential research design including qualitative, comparative and documentary studies (Fig. 1). The stages/phases were as follows:

*Phase one:* The contents of the resolutions of Iran’s Supreme Council of Health and Food Security between 2003 and 2017 were initially reviewed to determine the level of community participation in the health policy-making process. Additionally, the contents of all statements concerning food and nutrition policies were thematically extracted and categorized into four category (Fig. 3). These policies were provided to the participants during the qualitative interviews conducted as part of the study. The interview questions were designed with the aim of determining the process of problem identification and inclusion in the agenda, design, implementation and monitoring of policies, as well as the role of the people in each of these stages. This study was conducted in 2018 in Tehran, and the data were collected through semi-structured interviews. Targeted sampling was applied, and the interviews continued until data saturation was reached. A total of eight interviews were conducted with participants, including informed policy-makers, experts and representatives of NGOs active in the field of food and nutrition. The participants shared their perspectives on methods for including policies on the agenda and the status and voice of the public in the policy-making cycle. Utilizing a conceptual framework adapted from IAP2, referred to as the “participation spectrum” in this study, directed qualitative content analysis was employed to manage and analyse the data [21]. The participation spectrum was utilized to report the level of the community participation in the context of Iran. According to IAP2, the public participation level falls into various categories. According to Arnstein, participation encompasses a spectrum that ranges from non-participation to citizen power and is classified into eight levels and three categories, including “non-participation”, “tokenism” and “citizen power” [22]. In this study, the Spectrum of Public Participation developed by the International Association of Public Participation (IAP2; 2007) was utilized, which specifies the five levels of collaboration between the government and public as well as the expected outcomes: inform, consult, involve, collaborate and empower [23] (Fig. 2). “Inform” is the lowest, and “empower” is the highest level on this spectrum.

*Phase two:* To determine the optimal status for community participation, the upstream documents were reviewed. Moreover, to learn lessons from the challenges faced and the strategies employed by some similar countries to promote community participation in health policy-making, a comparative study was conducted to identify the best and most tailored practices of successful and pioneering countries in the field. Moreover, in an endeavour to contextualize the study within the larger social, cultural, political and economic context of post-revolutionary Iran, a qualitative study was designed to explore the perspectives of domestic policy-makers, managers and experts.

### Review of upstream documents

Data were collected through a review of the Constitution of Iran and other related upstream documents regarding general health policies. The Constitution is the peak of the legislative pyramid of Iran affecting the mid-level policies and legislations [20].

Using the known keywords in the field of community participation, including “participation”, “interaction”, “council”, “public cooperation” and “public consultation” and the two keywords “health” and “healthcare,” the content of the Constitution, legislations governing the first to sixth development programs and general health policies were reviewed. Beyond counting the frequency of words, efforts were made to determine the application of such keywords within the context of the legislation. Cumulative qualitative content analysis was used to analyse the data.

### Comparative study

A realist evaluation of a multi-case study of social participation in health system policy-making was conducted. Purposeful sampling was applied to select the countries. Five countries, namely Iran, Thailand, Tunisia, Chile and France, which have experienced community participation in health policy-making, were selected according to specific criteria. The data were extracted through a document review, including articles and reports published for each country. A realist approach was utilized for data analysis. According to this approach, an intervention produces outcomes through the creation of mechanisms in specific fields. These interactions are identified as a mechanism including context, intervention, mechanism and outcome (CIMO). The details of the methodology applied in this part of study are published elsewhere [24].

### Qualitative study

A study with a qualitative content analysis approach was designed and implemented between March and August 2022. Sampling continued until data saturation was reached. A total of 18 policy-makers, managers or experts who were directly or indirectly involved in health decision-making and were familiar with the community participation approach in the fields of health and sociology were included in the study. The data were collected through semi-structured interviews. This study received ethical code from Tehran University of Medical Science, and all participants provided verbal consent before interview or group discussion. During the interviews, in addition to questions addressing the importance of mobilizing community participation and its desired level on the spectrum, the interviewees’ opinions on facilitating and inhibiting factors, as well as strategies to promote community participation in Iran’s health system, were sought. To analyse the data, the conventional content analysis method was used. First, the recorded interview contents were transcribed word for word, and then the transcriptions were reviewed several times. Field notes were also entered into the analysis. Qualitative data were reduced to semantic units. Then, the semantic units were coded after several reviews by the researchers and according to their explicit meaning. The codes were compared in terms of similarities and differences and were categorized into subthemes and themes. Agreement on the codes, subthemes and themes was reached by the research team upon the review of the results. To increase the validity of the data, participant diversity, member checking and attention to negative cases were considered. The details of the methodology applied in this part of the study are published elsewhere [25].

*Phase three:* Using the results of the qualitative and comparative studies, suitable interventions to improve the participation level were extracted and categorized. Four policy options were formulated and presented for the structure required to implement health interventions. These options were discussed and studied in a policy dialogue session attended by seven experts and policy-makers. At the beginning of the meeting, a policy summary was presented to the participants. The advantages, challenges and feasibility of each option were introduced to the participants. Then, each participant had the opportunity to discuss and present their opinion. After the discussion, a survey form including three criteria, namely “impact on mobilizing community participation in health policy-making”, “operational feasibility” and “acceptance from the perspective of policy-makers,” was presented to the participants to score on a 5-point Likert scale from 1 to 5. The forms were collected upon completion. One participant took notes, and the meeting was recorded. The recording was transcribed, the participants’ opinions were reviewed and key points were extracted. The interventions and options were reviewed and discussed in a meeting with the representatives of non-governmental organizations, the House of Public Participation for Health, the National Healthcare Network, Urban and Rural Islamic councils and health policy-makers. Recruitment of representatives, in addition to the representatives of the non-governmental organizations active in the field of health, was based on purpose and topic and the environmental context of each policy option.They were invited by the head of the secretariat of the Supreme Council of Health and Food Safety. A total of 12 representatives participated in a 3-h meeting. At the beginning of the meeting, a policy summary was presented to the participants. Then, interventions and policy options were introduced to the participants. The participants discussed the advantages and challenges of each option in two groups of six. One facilitator was present in each group, and Chatham House rules were observed. After 90 min of discussion, the groups presented a summary conclusion. One study member took note of the points presented by the participants. After the discussion, a survey form including three criteria, namely “impact on mobilizing community participation in health policy-making”, “operational feasibility” and “acceptance from the perspective of policy-makers,” was presented to the participants to score on a 5-point Likert scale from one to five. The forms were collected upon completion. The survey forms were scored by a total of 19 stakeholders, including public representatives, policy-makers and experts. The Kruskal–Wallis test was used for statistical analysis.

Policy summary and survey form are available as an appendix (Additional file 1).

## Results

Since the main objective of the study was to determine the status quo and desired level of community participation in health policy-making in Iran and to provide relevant interventions to realize the desired level of participation, the findings of the study were categorized on the basis of these objectives.

### Status quo of community participation in Iran’s health policy-making

In all, 42 resolutions of the Supreme Council of Health and Food Safety related to food and nutrition during 2003–207 were categorized in four general groups, including “promotion of nutrition culture and literacy”, “promotion of cooking oil quality and reduction of fat consumption”, “provision of food security” and “food safety”. The level of public participation in each category was evaluated (Fig. 3).

Based on study findings, problem identification, prioritization and formulation of policies on food and nutrition were based on the evidence produced, policy-makers’ discretion and national resources. Community participation was not mobilized in these stages of policy cycle. In some policies, including reduction of trans-fatty acids and the elimination of baking soda in bakery products, community participation was mobilized in the policy implementation and policy evaluation phases at the level of informing (Table 1).

**Table 1:** Status quo of community participation in formulating food and nutrition policies in Supreme Council of Health and Food Security – Iran’s health system (*n* = 8)

Policy process	Level of public participation (IAP2)					
	No participation	Inform	Consult	Involve	Collaborate	Empower
Problem identification	+(8)	–	–	–	–	–
Agenda setting	+(8)	–	–	–	–	–
Policy formulation	+(8)	–	–	–	–	–
Policy implementation	+(6)	+(2)	–	–	–	–
Policy evaluation	+(6)	+(2)	–	–	–	–

### Desired community participation level in Iran’s health policy-making and essential interventions

#### Upstream legislations and documents

In various articles of the Constitution of Iran (Table 2) and Iran’s general health policies, the desired level of participation in the health system has been considered at the empowerment level according to the IAP2 model. In the legislations of the first to sixth development plans, as mid-level regulations in the legislative pyramid of Iran, provisions are not made to achieve the desired level.

**Table 2:** Contents of constitutional articles on community participation to realize people’s right to health

Article #	Relevant content in resolution	Key content
3	Community participation in determining their social destiny	Empowerment and individual participation
6	National affairs based on public opinion Members of the Islamic Consultative Assembly	National-level participation Presence of representatives in decision-making sessions Council members
29	Right to access health and medical services Revenues from community participation	Determination of public contribution to financing healthcare
43	Spiritual, political and social self-improvement Active participation in leadership Skills and innovation promotion	Public empowerment
58	Presenting legal proposals made at the suggestion of the members of the Islamic Consultative Assembly	Right to participate in policy cycle Participation through parliament representatives National-level participation
84	Each representative being accountable to the whole public The right to express opinion in all affairs	The right to participation at the national level Participation through parliamentary representatives
100	Expedited implementation of health programs through community participation Management of the affairs of each village, district, city, count, or province with the oversight of a council elected by the people	Participation through councils and elected representatives Participation at local and regional levels

#### Comparative study

The results of the comparative study indicated that some countries, including Thailand and France, have included community participation in their health policy system through the Health Assembly and the Health Conference. A wide range of health actors are involved in these structures. In Thailand, community health issues are identified at the local level and shared with the National Health Assembly for final review and decision-making upon review of local and regional committees. The representatives of public parties and vulnerable groups, patient associations and labour unions of France are members of the Health Conference and provide their advisory opinions to the government and the Minister of Health.

In Chile, Iran and Tunisia, measures have been taken to mobilize community participation in health policy-making. Tunisia is in a level higher than “inform”, but due to lack of a systematic perspective on this approach and application of inadequate and interrupted interventions, it has not yet been institutionalized in the health policy system. One of the major challenges in these countries is the nature of governance and the political and economic philosophy governing the society. The details of the results obtained in this part of study are published elsewhere [24]. Table 3 shows the CIMO for each selected country.

**Table 3:** CIMO of selected countries

Country	CIMO
Thailand	Raising the public voice in health policy (O); paradigm shift in health definition, dialogue space, political will and commitment, and trust (C); PHA and NHA (M); supportive legislation, expanding CSOs’ networks, developing required structures and public representatives in decision-making committees (I)
Iran	Creating socialization of health dialogue in policy-making space(O); health inequality, health transformation plan, political will (C); PHA and NHA (M); establishing Deputy for Social Affairs in MoHME (I)
Chile	Raising the patient voice in health service and strengthening monitoring of implementation of health policies (O); mobilization of popular classes, bio-psychosocial approach in primary healthcare (C); civil society and citizen councils, campaigns, and physical and online offices (M); legislation on the rights and duties of patients, strengthening of civil society (I)
France	Raising the patient (recently: public) voice in health service (O); civil society activism, dialogue space, political will and commitment, trust (C); CRSA and CNS (M); supportive legislation, creating required structures, patient representatives in decision-making committees (I)
Tunisia	White Book for Better Health in Tunisia (O); social dialogue space (C); regional meetings and national conference (M); right to health in the constitution, citizen juries (I)

(C), context; (I), intervention; (M), mechanism; (O), outcome; PHA, province health assembly; CSO, civil society organizations; CRSA, Conférence Régionale de Santé et de l'autonomie; CNS, Conférence Nationale de Santé (French National Health Conference)

#### Qualitative study

Based on the findings of the qualitative study, factors inhibiting community participation in Iran’s health system were classified into two themes, namely “government” and “community”, and the facilitating factors were grouped into a theme category, namely “institutionalization”. The present context in Iran, when viewed from the perspectives of participatory culture, economic, political and social conditions, has impeded the establishment of the necessary collaborative space between society and the government for effectively managing public affairs, including healthcare. From a health governance perspective, management changes within the executive context of health, a weak development-oriented view, a weak political will and the absence of a specific structure were identified as inhibiting factors hindering the formation and strengthening of collaborative processes. Fostering suitable socio-cultural and economic grounds, effective use of existing structures and mechanisms, and capacity-building through education are among the facilitating factors that can institutionalize community participation in health.

### Proposed solutions to improve community participation in health

**Fig. 4:**
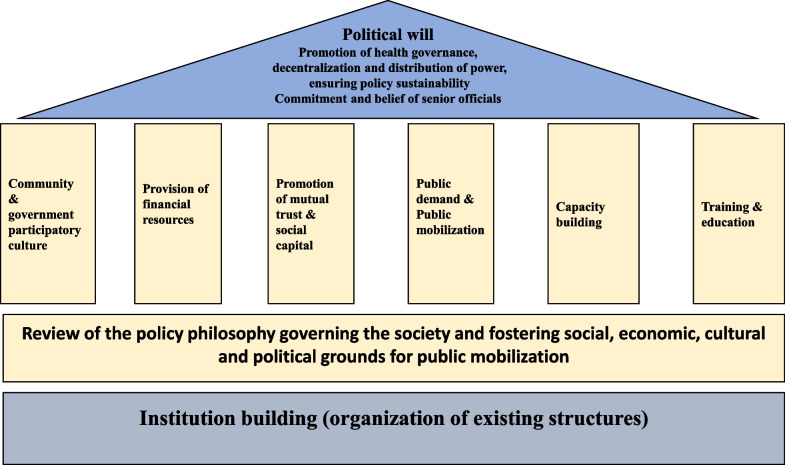
Conceptual model for institutionalizing public participation in health policy of IRI

To promote community participation in health, it is essential to implement a series of interventions in a supported social, economic, political and cultural framework, within a specific structure and institution. These interventions can be divided into four groups, including educational, process, structural and cultural interventions. Fostering a sense of responsibility among society members towards each other; capacity-building and training for both the community and government employees; establishing mechanisms for evidence and health data production that are comprehensible to society and policy-makers; population organization at the local and regional levels, education and raising awareness in the field of health; and participation in advocating for public health are among these interventions. Simultaneously, building mutual trust and promoting social capital can facilitate the creation of a conductive environment for interaction and dialogue between the society and government (Fig. 4).

Meanwhile, the presence of “political will” to promote a healthy governance hierarchy in the country, a shift in the prevailing political philosophy governing society, decentralization and equitable distribution of power, commitment, trust in senior officials and ensuring the sustainability of participation policies are key driving factors for the development of necessary interventions and the creation of suitable conditions and spaces for dialogue.

### Policy options to foster proper structures to promote community participation in health policy-making

Based on the results of comparative and qualitative studies, four policy options were extracted as described in Table 4. These options were determined on the basis of two bottom-up and top-down approaches to identify health sector issues, different environmental contexts and the position of the Secretariat of the Supreme Council for Health and Food Security.

**Table 4:** Policy options to foster proper structures to identify health problems and propose solutions

Option	Approach	Environmental context	Key official Health and Food Security Supreme Council secretariat
First	Bottom-up	House of Public Participation for Health and Health Assembly	Under the supervision of the MOHME or Office of the President
Second	Bottom-up	National Healthcare Network	Under the supervision of the MOHME
Third	Bottom-up	Urban and rural Islamic councils	Under the supervision of the Planning and Budget Organization or Office of the President
Fourth	Top-down	House of Public Participation for Health, health assembly, or healthcare network	Under the supervision of the MOHME

In the bottom-up approach, health issues are identified by the local community. Once a strategy is adopted, policy-makers seek a suitable approach in collaboration with the local community. In contrast, the top-down approach involves policy-makers identifying health issues and subsequently implementing strategies to address them. These contexts, in fact, pertain to the existing structures within Iran’s administrative and healthcare systems. It is feasible to institutionalize community participation within the healthcare sector by leveraging these established structures. Each of these structures has its own set of advantages and disadvantages, which were discussed during policy dialogues and community meetings. One notable advantage of the Rural and Urban Islamic Councils is that they are established in accordance with existing regulations, thereby receiving legal system support. Nevertheless, owing to their underperformance over the past 25 years since their establishment, some participants have voiced criticism regarding the council’s suitability for such efforts. In contrast, the health network enjoys recognition among society, particularly in rural areas, where trust in this structure is prevalent. Consequently, the likelihood of participation and cooperation within this framework is higher. However, the advantage of the health network has sparked considerable debate among some participants due to rural depopulation resulting from urbanization. Additionally, the House of Public Participation for Health and the Health Assembly have a shorter history, but they were still considered and discussed during the meeting by some participants due to their potential for inter-sectorial cooperation.

According to Table 5, no difference was observed between different options for “acceptance by policy-makers” and “operational feasibility”. In terms of the “impact on mobilizing community participation”, the first options, based on the environmental structure of the House of Public Participation for Health, and the second option, based on the environmental structure of the healthcare network, which had a bottom-up approach, had statistically significant differences with the fourth option with the same environmental structures but a top-down approach.

**Table 5:** Comparison of policy options for the appropriate structure to implement community participation interventions [Kruskal–Wallis’s test (*n* = 19); median (IQR)]

Criterion	Bottom-up approach			Top-down approach	*P*-value
	Option 1	Option 2	Option 3	Option 4	
Effect on community participation	4 (2)	4 (1)	3 (2)	3 (3)	0.02
Operational feasibility	3 (3)	3 (3)	2 (3)	4 (1)	0.13
Acceptance by policy-makers/society	3 (2)	4 (4)	2 (2)	2 (2)	0.08

IQR, inter quartile range

A key factor in the selection of options was the approach rather than the context. All participants unanimously favoured options based on a bottom-up approach. According to the participants’ votes in this study, when considering acceptance criteria from both policy-makers and the community, as well as operational feasibility, no statistically significant difference was observed. Thus, no structure was deemed preferable over the other in these aspects. However, when evaluating the effectiveness of community participation, the first option, which employed a bottom-up approach within the context of health assemblies and the House of Public Participation for Health, and the second option, also using a bottom-up approach within the framework of healthcare networks, showed a statistically significant difference from the fourth option, which shared the same context but adopted a top-down approach. This suggests that structures within the Ministry of Health and Medical Education (MoHME) were recommended over the urban and rural Islamic councils, despite the latter having legal support but falling under the Ministry of Interior.

## Discussion

Community participation is a recognized and promising approach to promote health and reduce health inequalities. Civil organizations can enhance the delivery of healthcare services by fostering dialogues between the government and citizens regarding health priorities, performance and accountability.

In this study, interventions adopted by the leading countries to strengthen participatory health governance were locally adapted according to the socio-cultural context of Iran. Hence, the result of this study can be effectively utilized by the policy-makers and health custodians for decision-making. One limitation of the study was the small sample size used to prioritize policy options due to low participation in the dialogue session. However, the study’s validity was enhanced by diversifying participants from various environmental structures.

In this study, “political will for action” emerged as the driving factor to institutionalize participatory health governance. It serves as a catalyst for promoting health sponsorship in the governance hierarchy, ensuing policy sustainability to mobilize participation, and delegating the decision-making power to the macro, meso and micro levels. It is worth noting that this fact necessitates a shift in the policy philosophy paradigm that governs society, fostering a foundation for dialogue and interaction.

“Political will” is a widely recognized key factor in countries with experience in participatory governance in health. Thailand and France, for instance, recognize it as a facilitating factor for community participation [10, 14], whereas its absence has hindered progress in Tunisia [26]. The World Health Organization also highlights political will as an essential factor in community participation in health [7].

“Creating structures” was identified as another intervention. The presence a proper structure not only acknowledges the participatory approach but also operationalizes and institutionalizes it through the implementation of various interventions. According to the findings of this study, Thailand has established the National Health Assembly of Thailand [10], and France has established the National Health Conference of France. [14] The analysis of data regarding the restructuring of the councils in Brazil indicates that there were not sufficient structural conditions to support decision-making and the monitoring of health activities and services. The lack of budget allocation, an executive secretariat and dedicated headquarters are constraints that limit health council members’ performance in monitoring and controlling health policies. [13].

“Mutual trust”, manifested through enhanced social capital and accountability and transparency mechanisms, was identified as another crucial factor in promoting the level of participation. In Thailand, social networks, as a key component of social capital, encourage and empower Thai citizens to participate in public affairs [27].

Other studies have also recommended that governments committed to effective and sustainable social participation should create an environment of trust for all stakeholders involved [28], coupled with a deep understanding of power dynamics and their influence on participatory processes [29]. Therefore, to promote community participation in the health policy system of Iran, it is essential to promote the belief in social participation in the national governance hierarchy.

This study identified “capacity-building” as one of the interventions at two levels: the community and government. At the community level, capacity-building encompassed the promotion of a participatory culture and spirit, empowerment, training, awareness-raising, skill development and addressing demands.

In the health promotion glossary of the World Health Organization, “empowerment” is defined as building the capacity to develop a strategy to mobilize stakeholder participation in identification of needs, decision-making and [taking] political, social and cultural actions to address such needs [30]. Kapiriri et al. defined “civic empowerment” as achieving self-confidence and self-belief to address social needs by acquiring or increasing the ability to demand social rights [29].

In this study, skill training, including the principles of negotiation and expression, respect for the opinion of others, acceptance, and effective criticism, was among capacity-building interventions identified at the community level. The World Health Organization considers public audience speaking as one of the main components of communication skills in capacity-building. In Madagascar, community representatives are invited to attend workshops since it is generally felt that they lack sufficient speech delivery skills, or in Portugal, members of the health council have expressed concerns about delivering their first civil society speech in front of a highly hierarchical official audience [7].

Other studies suggest that the capacity to interact with other stakeholders is vital for meaningful participation in social participation processes. These skills lead to the transformation of perceived inequalities into well-formulated arguments and explanations for actions [28, 31, 32].

This study also identified “organization of the local population” as an intervention. Within this concept, sub-categories included creating participation opportunities by determining mechanisms and methods for selecting public representatives, organizing NGOs through networking and establishing local development hubs.

One notion constantly emphasized by stakeholders in the process of participatory governance and social participation is representation of participating individuals [33–36]. This concept is closely linked to legitimacy and credibility. When participating individuals truly represent the perceived group, often the public, they inherently possess the necessary legitimacy to act as representatives. More importantly, the outcomes of such participatory processes are perceived as legitimate and legal [7].

Finally, due to the presence of multiple players, diverse structures and various legislations in the health policy environment and the efforts of pharmaceutical companies, insurance companies, some practitioners and policy-makers to maximize their economic and political interests, it is crucial to resolve the conflicts of interest of various actors in the health governance context. This might be explained by political economy approach to ensure the impact of the public demand on health policies. Political economy analysis involves examining the interplay between political and economic processes within a society. It encompasses the distribution of power and wealth among various groups and individuals, as well as the processes that establish, maintain and transform these relationships over time [37].

In the final section of this study, considering the significance of institutionalization and the establishment of specific structures to implement the identified interventions, policy options were presented. The first option, which employed a bottom-up approach within the context of House of Public Participation for Health and health assemblies, and the second option, also using a bottom-up approach within the framework of healthcare networks, showed a statistically significant difference from the fourth option, which shared the same context but adopted a top-down approach. This suggests that, regardless of the context chosen, the approach itself plays a paramount role in mobilizing public participation.

In terms of feasibility, half of the participants rated the fourth option, which employed a top-down approach, with a score of 4 or higher. Given that this approach represents a continuation of the current process, it is expected to encounter less resistance during implementation.

In terms of acceptance by policy-makers and society, half of the participants gave a score of 4 or higher to the second option, which utilized a bottom-up approach to identify health problems within the context of the health network. This can be attributed to the fact that this structure has been familiar to policy-makers and the public, especially in relevant villages, for the past 40 years. During meetings with public representatives, despite high confidence in the effectiveness of House of Public Participation for Health structures in mobilizing participation, their unfamiliarity to both the public and policy-makers was identified as a significant challenge.

Overall, it can be concluded that, according to the participants in this study, the bottom-up approach is of paramount importance in mobilizing public participation, regardless of the specific context.

Given that, in Thailand and France, the existing partnership structure enables these bodies to provide advisory opinions to the government and the Ministry of Health, it becomes essential for key individuals in charge to establish a direct relationship with the Ministry of Health, irrespective of their official positions. It is worth mentioning that some participants in the qualitative study believed in promoting the status of the secretariat in the national governance hierarchy.

## Conclusions

The contexts of the countries, from the perspective of participatory culture, along with economic, political and social conditions, create the essential collaborative space between the society and governments. In the presence of such contexts, the establishment of specific structures leads to the creation of the necessary mechanisms to improve the level of community participation and institutionalize participatory health governance. Simultaneously, the presence of political will at the highest levels of governance and the commitment of senior officials will serve as facilitating and driving factors. This transformation can occur through the modification of the political philosophy that governs the society. However, from a political economy perspective, effectively managing conflicts of interest among various actors in the health governance context is crucial for promoting community participation in health decision-making. Consequently, in Iran, establishing sustainable political will for participatory health governance and effectively managing conflicts of interest in this sector will facilitate the implementation of the identified interventions within an official structure and institution. In this scenario, the long-term institutionalization of the community participation approach within the health decision-making system is anticipated, fostering optimism for the effectiveness of health policies that take into account the public’s perspective. By harnessing the capacity of existing structures, particularly the healthcare network and House of Public Participation for Health, it becomes feasible to organize systematic health assemblies with a bottom-up approach to identify health problems and provide suitable solutions to address them.

### Supplementary Information

**Additional file 1.** Policy Summary of Community Participation in Health Policy-Making in Iran.

## Data Availability

The data are available by communicating authors.

## Abbreviations

IAP2: International Association for Public Participation
SDH: Social determinants of health
PHC: Primary healthcare
MoHME: Ministry of Health and Medical Education
NGO: Non-governmental organization
CIMO: Context, intervention, mechanism and outcome
IRI: Islamic Republic of Iran
