# Supplementary material for: Sustainable political commitment is necessary for institutionalizing community participation in health policy-making: Insights from Iran
Source: Health Res Policy Syst. 2024 Feb 13;22:23. doi: 10.1186/s12961-024-01111-z (PMC10863295; doi:10.1186/s12961-024-01111-z)
Supplement: Supplementary file 1 — Additional file 1. Policy Summary of Community Participation in Health Policy-Making in Iran. [file 12961_2024_1111_MOESM1_ESM.docx]

**In the Name of God**

**Policy Summary of Community Participation in Health Policy-Making in Iran**

**Introduction:**

One of the primary health care principle as proposed at the Alma-Ata Summit in 1978, was focus on community participation to improve health (1). Since then, effective measures have been taken to enhance and promote public and community participation in health care (2). Health policy makers have recognized the necessity of due consideration of public needs and views to ensure development of relevant policies (3) and to engage community participation which has been recognized as a health priority at global level to achieve sustainable development goals, to realize universal health coverage (4), implementation of preventive strategies, promotion of health, particularly among underdeveloped groups of the society and to control chronic diseases (5). Morgan believes: Effective participation of communities, having a positive effect on social capital, results in improved community empowerment and reduced inequalities in health (6). Meanwhile, Hadane recommends caution to consider empowerment as an outcome indicator for social participation interventions. (7)

In Iran, due consideration of community participation is based on the Constitution and the regulations based on which the Ministry of Health and Medical Education has been founded. This very fact has been emphasized in general health policies. However, the methods of promoting effective community participation in decision-making process are yet unclear. The question is, how can Iranian citizens be involved in the process of health policy-making?

The policy options as extracted from the study “Study the status of Iranian Community Participation level in health policy making, provide improvement policy options and assessment the policy maker's palatability" are to improve the level of community participation in the development of Iran's health policies to receive your valuable opinion.

Various theories are available on the levels of community participation; in health studies the Ornstein ladder model is applied which is based on power balance. According to its simplified form, 5 levels of community participation are identified.

1. Inform 2. Consult 3. Involve 4. Collaborate 5. Empower

**Taking time to fill this questionnaire is highly appreciated**

**Dear contributor/participant;** It would be highly appreciated if you could give a score of 1 to 5 to the criteria as stated in Table 2 according to the features presented in the policy options developed based on global evidence and national experts opinions (Table 1) and the considerations as presented for every option (Table 4).

**Kindly note your years of experience in policy-making or involvement in community based activities.**

**Policy-making experience of ………….years Community based activity of ……..years**

Table 1. Policy options to promote community participation in Islamic Republic of Iran

| **Option** | **Approach** | **Context** | **Focal Point**  **Secretariat of the Supreme Council of Health and Food Safety** |
| --- | --- | --- | --- |
| **First** | Bottom up | Current structure of public participation centers | Under MoH or Presidential Administration of Iran |
| **Second** | Bottom up | National Health Network Structure | Under MoH |
| **Third** | Bottom up | Urban and rural Islamic Council structure | Under Planning & Budget Organization or Presidential Administration of Iran |
| **Fourth** | Top to Bottom | Current structure of public participation centers or national health network | Under MoH |

Table 2. Evaluation criteria to prioritize policy options

| **Option** | **Effect on public participation in policy-making**  **(1-5)** | **Institutionalization Capacity**  **(1-5)** | **Acceptance by community representatives**  **(1-5)** |
| --- | --- | --- | --- |
| **First** |  |  |  |
| **Second** |  |  |  |
| **Third** |  |  |  |
| **Fourth** |  |  |  |

In case you have another option in mind, please note in table 2.

Table 3. Options recommended by participants in policy dialogue to promote community participation in health in Islamic Republic of Iran

| **Option** | **Approach** | **Context** | **Focal Point** |
| --- | --- | --- | --- |
|  |  |  |  |
|  |  |  |  |

Table 4. Implementation considerations of options as presented in Table 1

| **Option** | **International Evidence** | **Advantages** | **Challenges** | **Operational Feasibility** |
| --- | --- | --- | --- | --- |
| 1 | Similar to the model of Thailand and France | - Greater alignment of priorities with the needs of the local community - Benefiting from the capacity of NGOs at local and regional levels in identifying needs and prioritizing - Development oriented | - The need for capacity building and developing technical skills on the part of the government and the society - Restrictions imposed by the government on the number of NGOs active in the field and their interaction with the communities - This is the policy option of the former government, probably the health authorities would prefer to present a new plan. | There is the experience of National and provincial forum establishment. |
| 2 | Similar to Chile (social welfare services are also provided through PHC) | - Higher acceptance probability and faster implementation by MoH - Higher public trust due to involvement of social capital in the health sector - Greater horizontal transfer of information with the population under cover and health staff | - The need for capacity building and developing technical skills on the part of the government and the society - Health departments and network lack sufficient capacity and capability. - Possibility of SDH negligence and weakness in prioritization based on local community needs - Lack of internal consistency (weak cooperation between health department and the Council Secretariat) | Need to strengthen the network |
| 3 | Similar to the model of Thailand and France | - Legal capacity of the councils - Greater alignment of the priorities with the local community needs - Development oriented - Higher possibility to promote inter-sectoral cooperation | - The need for capacity building and developing technical skills on the part of the government and the society - Weakness in the performance of the Councils - Technical capacity weakness in the Planning & Budget Organization | Provincial governorates have higher implementing authority than the universities. |
| 4 | Similar to Chile (there is no institutionalized model, but measures are in place to monitor MoH policy implementation) | - Greater alignment of prioritize with policies and upstream document - Higher system acceptance | - The need for capacity building and developing technical skills on the part of the government and the society - Prioritize are not necessarily according to the local community actual needs. | Need to determine health indicators and their periodic evaluation |

**References:**

WHO. Declaration of Alma-Ata. Geneva: World Health Organization; 1978. http://www.who.int/hpr/NPH/docs/ declaration almaata.pdf. [Accessed 16 February 2009].

Odugleh-Kolev, Asiya & Parrish-Sprowl, John. (‎2018)‎. Universal health coverage and community engagement. Bulletin of the World Health Organization, 96 (‎9) ‎, 660 - 661. World Health Organization. [http://dx.doi.org/10.2471/BLT.17.202382](https://dx.doi.org/10.2471/BLT.17.202382)

European Observatory on Health Systems and Policies, Rajan, Dheepa, Brocard, Eva, Poulussen, Charlotte, Koch, Kira. et al. (‎2022) ‎. Beyond consultations and surveys: enhancing participatory governance in health systems. Eurohealth, 28 (‎1) ‎, 9 - 13. World Health Organization. Regional Office for Europe. <https://apps.who.int/iris/handle/10665/351077>

Marston C, Hinton R, Kean S, Baral S, Ahuja A, Costello A,et al.Community participation for transformative action on women’s, children’s and adolescents’ health. Bulletin of the World Health Organization.

2016; 94(376–382).

Rifkin SB. Lessons from community participation in health programs: a review of the post Alma-Ata experience. International Health. 2009; 1(1):31–6. https://doi.org/10.1016/j.inhe.2009.02.001 PMID:

24036293

Morgan LM. Community participation in health: perpetual allure, persistent challenge. Health Policy and

Planning. 2001; 16(3):21–230.

Haldane V, Chuah FLH, Srivastava A, Singh SR, Koh GCH, Seng CK, et al. (2019), Community participation in health services development, implementation, and evaluation: A systematic review of empowerment, health, community, and process outcomes. PLoS ONE 14(5): e0216112. <https://doi.org/10.1371/journal.pone.0216112>
